# Quercetin Nanoemulsion Ameliorates Neuronal Dysfunction in Experimental Alzheimer’s Disease Model

**DOI:** 10.3390/antiox11101986

**Published:** 2022-10-05

**Authors:** Nouf K. Alaqeel, Mona H. AlSheikh, Mohammed T. Al-Hariri

**Affiliations:** 1 Department of Biology, College of Science, Imam Abdulrahman Bin Faisal University, Dammam 34212, Saudi Arabia; 2 Department of Physiology, College of Medicine, Imam Abdulrahman Bin Faisal University, Dammam 34719, Saudi Arabia

**Keywords:** quercetin, nanoemulsion, Alzheimer’s disease, rats, immunohistochemistry, oxidative

## Abstract

Aluminum is the most abundant metal that can get admission to the human through several means that include our food, drinking water, cans, drugs, and deodorants, causing neurodegenerative diseases such as Alzheimer’s disease (AD). The present study aims to evaluate the role of quercetin nanoemulsion (QCNE) in attenuating neuronal dysfunction in aluminum chloride (AlCl_3_)-induced experimental AD. All animals were classified into six groups including negative control group (I): received a vehicle; QC group: received intraperitoneal (IP) injection of QC; Alzheimer’s group: received AlCl_3_ orally; treated group (I): received AlCl_3_ orally and IP injection of QC; treated group (II): received AlCl_3_ orally and QC orally; and treated group (III): received AlCl_3_ orally and IP injection of QCNE. At the end of the experimental period (30 days), the brain was used to study biochemical parameters (measurement of neurotransmitters (serotonin, dopamine, and norepinephrine), oxidant/antioxidant parameters (reduced glutathione, malondialdehyde, superoxide dismutase, and advanced oxidation protein product), and inflammatory markers (adiponectin, interleukin 1β, and plasma tumor necrosis factor-alpha)), while another part was for brain immune-histochemical analysis (study cyclooxygenases (COX-1 and COX-2)). Results showed that the mean value of oxidative stress markers was significantly increased in the AD group as well as the inflammatory biomarkers and all the study neurotransmitters, whereas these parameters were attenuated in treated groups, especially those that received QCNE. The immunohistochemistry findings confirm our results. Both approaches (QC and QCNE) succeeded in retracting the negative impact of AlCl_3_. Meanwhile, the effect of QCNE is more potent in mitigating the impact mediated by AlCl_3_ in treated animals. In conclusion, the treatment mainly by QCNE has huge potential in protecting against AlCl3-induced neuronal dysfunction, as shown in our results by the elevation of brain antioxidant/anti-inflammatory activities and neurotransmitter levels as well as mending of the histopathological changes in animal models.

## 1. Introduction

The frequency of neurodegenerative diseases significantly increases with age. Nowadays, the most distressing neurological progressive syndrome that appears in aged people is brain dementia [1]. Alzheimer’s disease (AD) is a complicated neuronal disorder and the most common cause of dementia. AD is characterized by advanced failure in language and memory in addition to damage to neurons and synapses, as well as selective exhaustion of neurotransmitter levels [2]. The main pathological changes of AD are the accumulation of intraneuronal Tau-protein tangles and extraneuronal plaque Beta-amyloid peptide [3].

Aluminum has been shown in previous studies to induce brain oxidative damage, neuronal degeneration, and amyloid deposition with subsequent cognitive deficits, which is similar to the pathogenesis of AD [4]. Moreover, aluminum is an environmental neurotoxin whose exposure has gotten increased due to lifestyle changes and has been implicated in the pathogenesis of AD [5]. However, as a possible mechanism of aluminum-induced neurotoxic potential, there is no evidence that aluminum may be the singular causative factor for AD but may act as a co-factor that can abet the development of the disease [6].

The treatment with an antioxidant agent is considered a suitable manner to decrease the risk of inflammation as well as neuronal dysfunction. Flavonoids are natural products that are found in vegetables, fruits, seeds, and nuts with high antioxidant properties. They are used to protect against several diseases, such as diabetes, cancer, liver disorder, atherosclerosis, and AD [7].

Quercetin (QC) is a flavonoid compound and has a wide range of health-related biological actions. QC is naturally present in many foods, including citrus fruit, apples, onions, parsley, sage, olive oil, grapes, chilis, berries, and bananas. Onions have the highest level of QC [8]. It increases immunity to viral infections [9], has anti-inflammatory effects [10], increases vascular elasticity by reducing atherosclerosis [11], improves blood glucose regulation [12], and has an anti-carcinogenic effect [13]. The latter is caused by inhibition of the tumor necrosis factor-alpha (TNF-α) in macrophages [14]. The anti-inflammatory properties of QC perhaps are by inhibiting the production of enzymes involved in inflammation (cyclooxygenase and lipoxygenase) [15]. It also inhibits the release of tryptase histamine and cytokines from mast cells through the inhibition of Ca^++^ influx [16]. It enhances vascular health by inhibiting vascular adhesion molecule 1 (VCAM-1) [17].

Additionally, QC has a high antioxidant property, so it is used as a talented treatment of different diseases. The antioxidant capacity of QC is related to its ability to diffuse through the cell membrane and sweep the free radicals. Thus, the structure of QC as a flavone (pentahydroxyflavone) facilitates the metal ions’ chelation through its phenolic structure; consequently, it scavenges alkoxyle and lipid alkoxyle radicals (LAR) [18].

However, QC has low solubility in water as well as in artificial gastric and intestinal juices, which limits its bioavailability upon oral administration [19], whereas the insolubility of this compound disturbs its applications consequently. Many approaches have been introduced to enhance the bioavailability of QC and enhance the absorption significantly as well as in crossing the blood–brain barrier, which is strongly affected by its solubility in the vehicles used for the oral administration of QC [20].

Nanoemulsion is an established system that has a submicron size of particles. The nanoemulsion system has been used to provide the active constituent. It can deliver the active component against different environmental conditions [21].

Nanoemulsions are more advantageous than other conventional delivery systems since their droplet sizes is below the micrometers range, so they easily pass the stringency of intravenous administration. As reported, the parenteral administration of nanoemulsions employed in natural and other bioactive compounds attests to the merits they possess over other systems, as their transit time, absorption, and efficacy are highly improved, while drug toxicity is reduced. Therefore, they are the perfect drug delivery systems for hormones, diuretics, antimicrobials, and steroids [22]. Accordingly, the nanoemulsion technique was used in order to enhance QC effects [23].

Although QC has been investigated extensively for its neuroprotective effects, there are still several limitations related to its oral bioavailability and aqueous solubility [24], which could be overcome through QC Nanoemulsion (QCNE) preparation.

Nevertheless, there is very little information regarding the effect of QCNE preparation in AD.

Thus, the aim of this study is to evaluate the potential role of QCNE in attenuating neuronal dysfunction in aluminum chloride (AlCl_3_) induced experimental AD. The therapeutic effects were evaluated by measuring levels of oxidative stress, inflammatory markers, and neurotransmitters in brain homogenates, as well as by histopathological examination of the brain tissue of all rats in all groups.

## 2. Materials and Methods

### 2.1. Materials

Chemicals: All HPLC standards (dopamine, norepinephrine, serotonin), AlCl_3,_ and QC were purchased from Sigma Aldrich Chemicals Company (St Louis, MO, USA). Palm-based ester (POE) and Tween 80 were purchased from RöhmPharma, Illertissen, Germany. Lecith was purchased from Lipoid, Germany. All other used chemicals were HPLC grade and found in our laboratory.

### 2.2. Methods

#### 2.2.1. Experimental Design

A total of 48 adult albino male rats (weighing 140 ± 10 g) were obtained from the animal house of Imam Abdulrahman Bin Faisal University (IAU). All rats were housed at the temperature range of 22 ± 2 °C, under a 12-hour light/12-hour dark cycle separately in individual suspended stainless steel cages, and before the experiment, they were allowed to acclimatize for a period of 7–10 days; animals were freely allowed to access food and water [25] and were divided into six groups (*n* = 8) as follows: negative control group (I): received a vehicle; QC group: received intraperitoneal (IP) injection of QC “15 mg/kg body weight/day” [26]; Alzheimer’s group (AD): received AlCl_3_ orally; treated group (I): received AlCl_3_ orally and during this period animals received IP injection of QC free nanoemulsion “15 mg/kg”; treated group (II): received AlCl_3_ orally and during this period animals received QC orally “15 mg/kg day” [27]; and treated group (III): received AlCl_3_ orally and during this period animals received IP injection of QCNE “15 mg/kg body weight/day”. The experimental protocol was approved by the ethical committee at IAU (IRB-2022-01-225).

#### 2.2.2. Preparation of Quercetin Nanoemulsion

One gram from QC was dissolved in 20 mL of palm oil ester (PEO) and 0.3 g lecithin; to this mixture, 10 mL deionized water containing 1 g Tween 80 were added dropwise. The final mixture was then homogenized “at 12 rpm for 10 min followed by sonication at 125 W in an ice bath for 50 min”. The resultant QCNE was then stored at 4 °C until used [28].

#### 2.2.3. Induction of Alzheimer’s Disease

Rats received AlCl_3_ (100 mg/Kg body weight/day) orally after dissolving in 0.9 sodium chloride at pH 7.4 for thirty days [29].

#### 2.2.4. Tissue Homogenate

At the end of the experimental period (30 days), rats were kept fasting overnight, anesthetized using 5% Sevoflurane with 95% of oxygen, and blood was collected from the inferior vena cava [30]. The brain was removed quickly and washed with ice-cold saline. The brain was divided into two parts: the first one was for biochemical parameters, and the second part was for immune-histochemical analysis. The brain was segmented into small pieces and homogenized according to the procedure mentioned somewhere else. Briefly, “the sample were homogenized in phosphate buffer at pH 7.4 and centrifuged at 1000 G using cooling centrifuge for 15 min at 4 °C, and the supernatant was removed for estimation of biochemical parameters” [31].

#### 2.2.5. Measurement of Brain Oxidative Stress and Inflammatory Markers

Brain oxidant/antioxidant parameters “reduced glutathione (GSH), malondialdehyde (MDA) and superoxide dismutase (SOD) were estimated by spectrophotometers as described before” [32]. In addition, brain advanced oxidation protein products (AOPP) were estimated according to Hoozemans et al. (2008) [33]. Moreover, bain adiponectin, interleukin 1β (IL-1β), and plasma TNF-α were estimated by ELISA in accordance with the manufacturer guidelines.

#### 2.2.6. HPLC Method for Neurotransmitters Estimation

Brain serotonin and the two catecholamines (dopamine and norepinephrine) measurements were carried out using HPLC, as described previously [34]. Reversed-phase (RP) column (C18, 25 × 0.46 cm i.d. 5µm) was used as a stationary phase, while the mobile phase consisted of potassium phosphate buffer and methanol in a ratio of 97/3 (*v*/*v*) at a flow rate of 1.5 mL/minutes and 270 nm; the concentration of each neurotransmitter was calculated from the corresponding standard curve.

#### 2.2.7. Immunohistochemistry of Brain Cyclooxygenases

The brain was embedded in 10% formalin; 5 μm thin sections were prepared on slides (positively charged). The sections were treated with 0.2% saponin for thirty minutes to remove the endogenous peroxidase; after that, hydrogen peroxide in methanol (3%) was added and left to react with 10% normal rabbit serum to block nonspecific reactions. Both of the polyclonal antibodies for cyclooxygenases (COX-1 and COX-2) (Thermo Fisher Scientific, Fremont Blvd, Waltham, MA, USA) were diluted and left to react with the prepared sections. The biotin-labeled anti-goat immunoglobulin G antibody was incubated at room temperature for 15 min to form a complex of streptavidin–biotin. After that, the sections were incubated for 10 min at room temperature with peroxidase-labeled streptavidin. The color was developed using diaminobenzidine (DAB) and then examined under the microscope with hematoxylin [34].

#### 2.2.8. HPLC Condition

In this method, acetonitrile, methanol, and phosphate buffer were used in a ratio of (25/10/965) v/v to prepare the mobile phase at a pH of 3.5. This mobile phase was filtered twice through a membrane filter (0.45 μm pore size) before running at a flow rate of 1 mL/ minute through a reversed-phase column (250 × 4.6, particle size 5 μL). An electrochemical detector was used for the detection with 600 mVcell potential.

#### 2.2.9. Statistical Analysis

The data were expressed as mean ± standard error (SE). One-way analysis of variance (ANOVA) was used in this study. Probability (*p* value) was considered significant when it < 0.05. The distribution of the data was verified to be normal using Tests of Normality (SPSS, version 26).

## 3. Results

**Figure 1:**
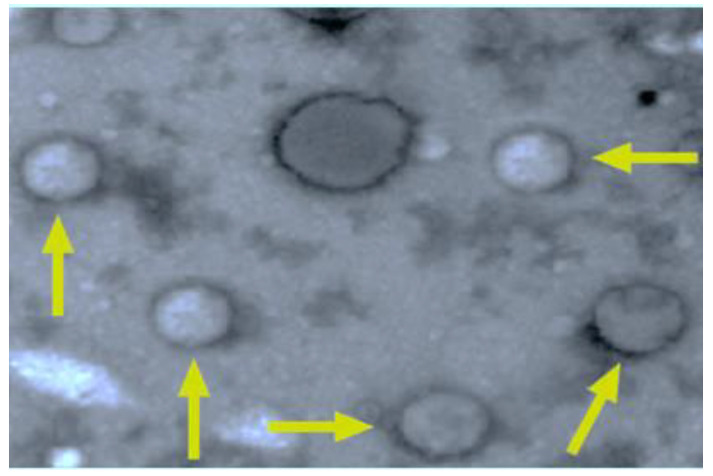
QCNE’ particles as appeared by transmission electron microscope.

As shown in Figure 1, one gram of the surfactant resulted in a spherical edge and a defined spherical shape with a smooth uniform surface as appeared in the transmission electron microscope (TEM).

In this study, the mean value levels of all parameters were insignificantly changed in the QC group compared to the control, indicating partial safety of QC being used.

In the treated groups (I, II, and III), the mean values of all parameters were insignificantly changed compared to the control group, representing the safety of the surfactant (tween 80).

**Figure 2:**
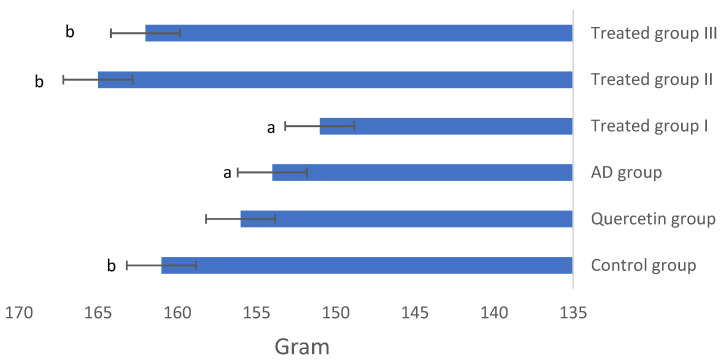
Body weight in different studied groups. a: Significant difference at *p* < 0.05. compared to the control group. b: Significant difference at *p* < 0.05 compared to AD group.

Figure 2 shows the mean changes in body weight in each group. Rats induced with AlCl_3_ (AD group) showed a significant decrease in body weight over the 30 days when compared with control rats. QCNE treatment to AlCl3-induced AD rats (treated groups II and III) significantly increased the mean body weight compared with the AD group to become near the control rats indicating the safety of the QCNE.

In treated group II, QC administration significantly increased brain SOD and GSH while decreasing the oxidation parameters (MDA and AOPP) compared to the AD group, whereas all these values were significantly changed compared to the control group. Additionally, the treatment with QCNE in group III showed the best results compared to QC alone (group II) to become near the control group, especially in SOD and GSH (Table 1).

Injection of AlCl_3_ not only increased oxidative stress but also increased the levels of pro-inflammatory markers, as shown in the present study. Thus, the mean value levels of IL-1β, TNF-α, and adiponectin were significantly increased in the AD group compared to the control (Table 2). As appeared in this current study, the QC administration significantly decreased inflammatory markers in treated groups II and III, whereas QCNE results appeared more sounded that became more or less near the results of the control group (Table 2).

**Figure 3:**
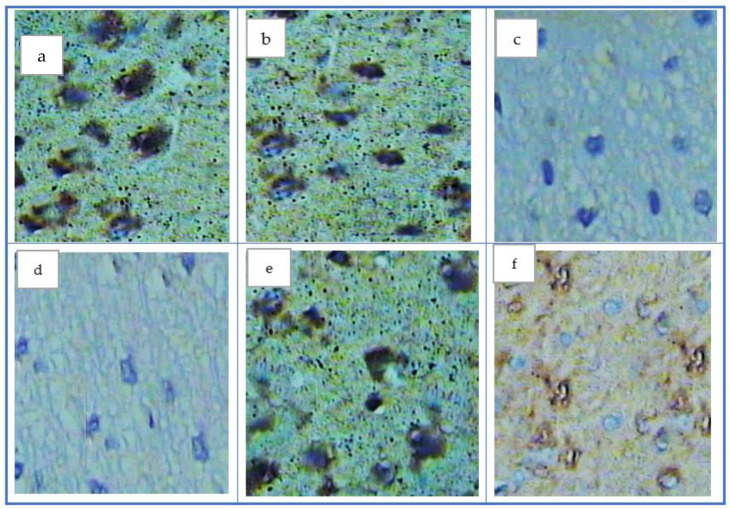
COX-1 in different studied groups, (**a**) control group appeared positive (brown patches in brain tissue, (**b**) quercetin group appeared as the same as the control group, (**c**) the AD group appeared to have negative reaction (disappearance of the brown patches), (**d**) the treatment group I showed disappearance of brown patches (**e**) the treatment group II appeared to have a positive reaction by appearance of the brown color. (**f**) treated group III appeared to have a positive reaction to become closed to the control group.

Evaluation of immune histochemical results of both the control and QC groups showed a positive reaction of COX-1, as demonstrated by the presence of a brown color (Figure 3a,b), while a negative reaction of COX-1 in the AD group appeared in the absence of a brown color (Figure 2c). In addition, treated group I showed a negative reaction—i.e., absence of the brown color (Figure 3d). Contrarily, treated group II that received QC showed a positive reaction of COX-1 as shown by a faint brown color (Figure 3e), whereas treated group III, which received QCNE, showed a strong positive reaction of COX-1 as shown by brown color which appeared more or less closed to the control group (Figure 3f).

**Figure 4:**
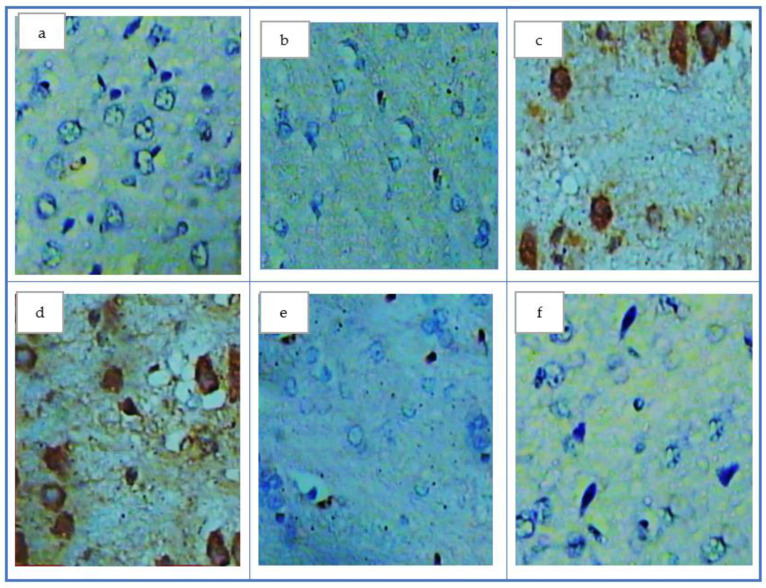
COX-2 in different studied groups, (**a**) control group appeared to have a negative reaction (absence of the brown patches in brain tissue; (**b**) quercetin group appeared as the same as the control group; (**c**) the AD group which appeared to have a positive reaction (appearance of the brown color); (**d**) the treatment group I appeared to have a positive reaction (appearance of the brown color); (**e**) the treatment group II appeared negative reaction by absence of the brown color; (**f**) Treated group III appeared to have a negative reaction to become more or less near the control group.

The examination of COX-2 in the control group and the healthy rats that received QC showed a negative reaction against COX-2, as appeared by the disappearance of the brown color (Figure 4a,b). However, the AD group and AD that received only the surfactant showed a positive reaction with COX-2, as demonstrated by the appearance of the brown color (Figure 3c,d). Both treated groups II and III appeared to have a negative reaction against COX-2, as visible by the disappearance of the brown color to return to the control group (Figure 4e,f).

The mean value levels of all neurotransmitters (norepinephrine, dopamine, and serotonin) as indicators of the disturbance of brain function were increased significantly in the AD group when compared to the control. However, in treated group II, QC significantly attenuated this elevation, and the mean value of neurotransmitters became significantly decreased compared to the AD group, although it was still significantly higher compared to the control. Nevertheless, administration of QCNE in treated group III significantly reduced these values compared to AD and insignificantly changed compared to the control. When comparing treated groups II and III, we found that QCNE significantly decreased neurotransmitter values compared to QC alone and that these values became close to the control group (Table 3).

## 4. Discussion

The AD experimental model in this study was induced by oral AlCl_3_ at a dose of 100 mg/kg daily for 30 days. The AD condition was confirmed by increased levels of oxidative stress biomarkers, an increase in inflammatory markers (COX-1 and COX-2), and a significant increase in levels of all neurotransmitters (dopamine, norepinephrine, and serotonin).

After inducing AD, QC was found to be effective in protecting against neurodegenerative changes. From another perspective, this study shows that novel insights into the surfactant (tween 80) in the preparation of QCNE in a dose of 15 mg/kg body weight/day for 30 days were very effective as anti-amnesic effects in reversing and ameliorating the neuronal degenerative changes, reducing oxidative stress and inflammatory biomarkers, as well as normalized the neurotransmitter levels associated with AD induced by AlCl_3_ in the experimental model; these findings are confirmed by immunohistochemistry of brain COX-1 and COX-2. This was in agreement with Rifaai R.A. and his coworkers in 2020, who found that QC nanoparticles reduced the damaging effect of AlCl_3_ on hippocampal neurons at the molecular, cellular, and subcellular levels [35].

Nowadays, nano-systems, such as nanoemulsion developments, have been given the highest priority in medical fields. QCNE preparation offered an electrostatic repulsion and a reduction of the surface tension that in turn, gave long-term physical stability to the produced emulsion [36].

Intriguingly, our findings validated the previously reported biological effects of QC in rats exposed to AlCl_3_. QC can have an anti-neuroinflammatory effect by downregulating the production of pro-inflammatory cytokines, as well as antagonizing cell toxicity by oxidative stress in neurons. Even after absorption, QC metabolites are methylated, sulfated, and/or glucoronate, and all have been shown to bear neuroprotection [1,37].

However, reported evidence showed that poor bioavailability and instability of QC are the main problems associated with the therapeutic index efficacy of QC [38,39,40]. Therefore, developing a new approach to QC delivery, such as nanoemulsion preparation, may be required to protect the active ingredient within the nanoparticulate network and thus prevent its degradation, thereby enhancing its bioavailability [41]. It also rarely poses any toxicity to normal cells [42]. Hence, the use of nanoemulsion is encouraged to enhance the therapeutic effectiveness of natural agents.

According to a recently published review, very few research studies were conducted on nervous system disorders using the nanoemulsion form of QC [43].

Evidence of oxidative stress in AD is manifested by a decrease in the antioxidant capacity and/or lipid peroxidation [44]. AOPP is one of the important indicators of the oxidative stress created in the neurons. Thus, oxidation of high as well as low molecular weight proteins occurred inside the brain, and that is called AOPP, which is considered a hallmark of oxidative stress [45].

The biomarkers were estimated in this study to give us a broad image of the role of antioxidative stress as well as the anti-inflammatory effects of QCNE in affecting brain health through turbulences in the neurotransmitter’s levels and brain tissues.

It was suggested that with the oxidation of protein molecules in AD, the expression of inflammatory proteins such as beta amyloid plaques is mediated and increases brain inflammation resulting in alteration of COX-1 and 2 that, in return, help in the appearance of AD symptoms [46,47].

When evaluated in terms of brain tissues antioxidant properties, QCNE showed the most powerful antioxidant effects.

Accumulation of AlCl_3_ is escorted by the release of cytochrome C from mitochondria, which results in an elevation of free radical generation and an increase in the production of pro-inflammatory cytokines. Furthermore, it increases the gene expression of TNF-α and IL-1β. Additionally, systemic toxicity induced by AlCl_3_ could be reflected by an increase in systemic inflammation changeability, such as IL-1β and TNF-α [48]. In further support of the present findings, QC has already been shown to inhibit neuroinflammation, which is the hallmark in the pathogenesis of AD [49], by decreasing the production of inflammatory chemokines and cytokines [50]. It is well reported that QC inhibits inflammatory gene expression in microglia and regulates the expression of COX-1 and COX-2 [1].

According to our results, QCNE succeeded in decreasing all study inflammatory marker (Adiponectin, IL-1β, and TNF-α) levels became close to the control group. Therefore, the potential anti-inflammatory effects of the anti-structure defect structure of the QCNR revealed the role of nanoemulsion form, which enhanced the effectiveness of QC and gave it more solubility and stability [20,43].

Administration of AlCl_3_ caused marked accumulation of aluminum in the brain tissues leading to structural and functional disturbance [51]. QCNE, as flavonoid compounds, are very effective metal ion chelators and form stable products with beryllium, aluminum, iron, and zinc ions [52]. The present results suggest that QCNE has chelating properties and can protect brain tissues.

The current management protocol for AD includes symptomatic treatment through repairing the levels of neurotransmitters involved [53]. Neurotransmitters are very sensitive to reactive oxygen species generation because of their nature as a protein (amino acids) [54], and the impairment of neurotransmitters induced by AlCl_3_ may be related to the capability of aluminum to increase inflammation and inhibit cholinergic projections [52,54,55], besides its ability to damage the membrane constituents such as lipid and proteins as well as the reduction of antioxidant enzymes [56]. These disturbances can alter the reliability of the neuron and eventually cause their disintegration, generating shortages in the concert level of knowledge and understanding.

The treatment of rats with the QC showed a significant increase in all the study neurotransmitter levels. According to the reported evidence, QC modulates the neurotransmitter levels by inhibiting their enzymatic degradation as well as affect positively on neurotransmission between neurons [57]. This is attributed to the polyphenol and flavonoid contents of QC, which have anti-inflammatory and antioxidant properties [58].

Finally, significant interest has been more focused on determining whether several natural products, known as nutraceuticals, may exert neuroprotective effects on the aging nervous system. QC has received the most attention in this regard [59]. Since QC can, in addition to what has been mentioned previously as neuroprotective pathways, stimulate autophagy, “cellular degradative pathways that involve delivery of the cytoplasmatic cargo to the lysosomes,” which is considered another potent neuroprotection mechanism for QC prevent neurotoxicity of beta amyloid 1-42 [1]. On such a basis, the superior efficiency of QC compared to other compounds could be explained [60].

In the same line, it has been reported that QC can flux into brain regions [61]. Additionally, it is important to mention that at the microvessel level, antioxidation and antiinflammation properties of QC would be added to improve blood flow and counteract the ischemic changes in the brain [62]. Therefore, it is possible that QC, with its beneficial biological properties, is able to penetrate the blood–brain barrier and can protect against neurotoxicity [63].

An interesting aspect, when various potent antioxidants (QC, Boldine, vitamin E and Trolox) were studied for their capacity to increase cell survival in the hydrogen peroxide-induced cytotoxicity, only QC and/or structurally similar flavonoids protected PC12 cells from the oxidative insult [64,65]. Moreover, reported data showed that the antioxidant and neuroprotective activities of QC were more efficacious than that of vitamin C and melatonin, respectively [63,66].

## 5. Conclusions

The treatment mainly by QCNE has a huge potential in protecting against AlCl3-induced neuronal dysfunction, as shown in our results, by elevating brain antioxidant/anti-inflammatory activities and neurotransmitter levels as well as mending the histopathological changes in animal models. This might open the gate for more studies on the therapeutic applications of QCNE in AD.

## 6. Limitations

This study lacks the behavioral neurocognitive and memory impairment measurement of AlCl_3_ or Quercetin and the pathological characterization of the histological hallmark of AD, namely amyloid plaque accumulation and neurofibrillary tangles.

## Figures and Tables

**Table 1:** Oxidative stress status indifferent studied groups.

Groups	SOD (U/g Tissue)	GSH (m mol/g Tissue)	MDA (nmol/g Tissue)	AOPP (ng/g Tissue)
Control group	320.23 ± 1.21	44.38 ± 0.67	11.34 ± 0.22	3.62 ± 0.65
Quercetin group	314.65 ± 0.98	45.32 ± 0.73	10.31 ± 0.16	3.42 ± 0.48
AD group	211.24 ± 0.93 ^a^	22.01 ± 0.59 ^a^	42.09 ± 0.74 ^a^	21.71 ± 0.59 ^a^
Treated group I	214.84 ± 1.10 ^a^	23.20 ± 0.61 ^a^	43.02 ± 0.53 ^a^	3.51 ± 0.65 ^a^
Treated group II	275.03 ± 0.85 ^a,b^	37.34 ± 0.68 ^a,b^	31.21 ± 0.22 ^a,b^	14.29 ± 044 ^a,b^
Treated group III	313.36 ± 0.76 ^b,c^	42.87 ± 0.80 ^b,c^	22.82 ± 0.41 ^a,b,c^	7.23 ± 0.67 ^a,b,c^

^a^ Significant difference when *p* < 0.05 compared to the control group. ^b^ Significant difference when *p* < 0.05 compared to the Alzheimer’s disease (AD) group. ^c^ Significant difference when *p* < 0.05 compared to the treated group II. GSH: reduced glutathione; MDA: malondialdehyde; SOD: superoxide dismutase; AOPP: brain advanced oxidation protein product (AOPP).

**Table 2:** Inflammatory markers in different studied groups.

Groups	IL-1β (Pg/mL)	TNF-α (Pg/mL)	Adiponectin μg/g Tissue
Control group	24.04 ± 0.99	26.83 ± 0.93	2.72 ± 0.23
Quercetin group	22.37 ± 1.34	25.71 ± 0.98	2.41 ± 0.25
AD group	57.32 ± 1.02 ^a^	56.28 ± 1.02 ^a^	8.72 ± 0.13 ^a^
Treated group I	56.63 ± 0.91 ^a^	57.11 ± 1.11 ^a^	8.43 ± 0.18 ^a^
Treated group II	43.22 ± 1.21 ^a,b^	40.12 ± 0.96 ^a,b^	6.50 ± 0.14 ^a,b^
Treated group III	31.01 ± 0.78 ^a,b,c^	28.02 ± 0.82 ^b,c^	3.12 ± 0.09 ^b,c^

^a^ Significant difference at *p* < 0.05 compared to the control group; ^b^ Significant difference at *p* < 0.05 compared to Alzheimer’s disease (AD) group. ^c^ Significant difference at *p* < 0.05 compared to treated group II.

**Table 3:** Brain neurotransmitters (µg/g tissue) in different studied groups.

Groups	Dopamine	Norepinephrine	Serotonin
Control group	4.12 ± 0.34	3.85 ± 0.23	3.11 ± 0.27
Quercetin group	4.03 ± 0.48	3.92 ± 0.26	3.23 ± 0.31
AD group	6.92 ± 0.37 ^a^	6.31 ± 0.28 ^a^	5.76 ± 0.25 ^a^
Treated group I	6.73 ±0.72 ^a^	6.33 ± 0.40	5.83 ± 0.32
Treated group II	5.93 ±0.60 ^a,b^	5.29 ± 0.24 ^a,b^	4.50 ± 0.30 ^a,b^
Treated group III	4.37 ± 0.36 ^b,c^	4.012 ± 0.41 ^b,c^	3.31 ± 0.46 ^b,c^

^a^ Significant difference at *p* < 0.05 compared to the control group. ^b^ Significant difference at *p* < 0.05 compared to Alzheimer’s disease (AD) group. ^c^ Significant difference at *p* < 0.05 compared to treated group II.

## Data Availability

Data are contained within the article.
